# Hydrogen in Drinking Water Reduces Dopaminergic Neuronal Loss in the 1-methyl-4-phenyl-1,2,3,6-tetrahydropyridine Mouse Model of Parkinson's Disease

**DOI:** 10.1371/journal.pone.0007247

**Published:** 2009-09-30

**Authors:** Kyota Fujita, Toshihiro Seike, Noriko Yutsudo, Mizuki Ohno, Hidetaka Yamada, Hiroo Yamaguchi, Kunihiko Sakumi, Yukiko Yamakawa, Mizuho A. Kido, Atsushi Takaki, Toshihiko Katafuchi, Yoshinori Tanaka, Yusaku Nakabeppu, Mami Noda

**Affiliations:** 1 Laboratory of Pathophysiology, Graduate School of Pharmaceutical Sciences, Kyushu University, Fukuoka, Japan; 2 Division of Neurofunctional Genomics, Medical Institute of Bioregulation, Kyushu University, Fukuoka, Japan; 3 Department of Oral Anatomy and Cell Biology, Graduate School of Dental Sciences, Kyushu University, Fukuoka, Japan; 4 Department of Integrative Physiology, Graduate School of Medical Sciences, Kyushu University, Fukuoka, Japan; 5 R&D Center, Home Appliances Manufacturing Business Unit, Panasonic Electric Works Co., Ltd., Osaka, Japan; University of Cambridge, United Kingdom

## Abstract

It has been shown that molecular hydrogen (H_2_) acts as a therapeutic antioxidant and suppresses brain injury by buffering the effects of oxidative stress. Chronic oxidative stress causes neurodegenerative diseases such as Parkinson's disease (PD). Here, we show that drinking H_2_-containing water significantly reduced the loss of dopaminergic neurons in PD model mice using both acute and chronic administration of 1-methyl-4-phenyl-1,2,3,6-tetrahydropyridine (MPTP). The concentration-dependency of H_2_ showed that H_2_ as low as 0.08 ppm had almost the same effect as saturated H_2_ water (1.5 ppm). MPTP-induced accumulation of cellular 8-oxoguanine (8-oxoG), a marker of DNA damage, and 4-hydroxynonenal (4-HNE), a marker of lipid peroxidation were significantly decreased in the nigro-striatal dopaminergic pathway in mice drinking H_2_-containing water, whereas production of superoxide (O_2_•^−^) detected by intravascular injection of dihydroethidium (DHE) was not reduced significantly. Our results indicated that low concentration of H_2_ in drinking water can reduce oxidative stress in the brain. Thus, drinking H_2_-containing water may be useful in daily life to prevent or minimize the risk of life style-related oxidative stress and neurodegeneration.

## Introduction

It has been reported that molecular hydrogen (H_2_) selectively reduces the hydroxyl radical, the most cytotoxic of the reactive oxygen species (ROS), and can thereby effectively protect cells. Thus, inhalation of H_2_ gas strongly suppressed ischemic and reperfusion brain injury [1], [2] and consumption of water saturated with H_2_ (H_2_ water) prevented stress-induced impairments in learning tasks during chronic physical restraint [3] by buffering the effects of oxidative stress or superoxide formation [4]. Oxidative stress, linked to mitochondrial damage, is also a primary cause of Parkinson's disease (PD) [5]–[7]. PD is regarded as an intractable neurodegenerative disease with pathological changes to dopaminergic neurons in the substantia nigra (SN) and nigro-striatal dopaminergic nerve terminals, leading to movement disorders such as tremor, rigidity and akinesia [8]. The 1-methyl-4-phenyl-1,2,3,6-tetrahydropyridine (MPTP)-based PD model has been important in elucidating the molecular cascade of cell death in dopaminergic neurons as well as the discovery of PD genes [9]. MPTP itself is not toxic, and as a lipophilic compound can cross the blood-brain barrier. Once inside the brain, MPTP is metabolized into the toxic cation 1-methyl-4-phenylpyridinium (MPP+) by the enzyme monoamine oxidase B (MAO-B) in glial cells. MPTP has a quite selective ability to cause neuronal death in dopaminergic cells, apparently through a high-affinity uptake process, through a dopamine transporter (DAT) [10], after it has been released from the glial cells. Inside dopaminergic neurons, MPP+ interferes with complex I of the electron transport chain, a component of mitochondrial metabolism, which leads to cell death and causes the buildup of free radicals, toxic molecules that contribute further to cell destruction [11].

Today, antioxidant compounds are widely recognized for the potential therapeutic treatment of oxidative stress diseases. Some antioxidant drugs and antioxidant materials in foods have been tested in the MPTP mouse model [12], [13]. Antioxidants not only in foods but also in drinking water would offer a great advantage over other forms of antioxidant therapy. In fact, it was reported that drinking electrolyzed H_2_-saturated water showed an effect in reduction of oxidative stress in rats, as measured by urine oxidized guanine and hepatic lipid peroxide [14]. Recently it was also shown that drinking H_2_-saturated water, instead of inhaling H_2_ gas, prevents cognitive impairment by reducing oxidative stress [3]. According to Nagata et al. [3], even in drinking water, H_2_ can be delivered to the blood in minutes.

Using the widely accepted PD model, we have tested the effect of H_2_-containing drinking water on the MPTP-induced loss of dopaminergic neurons. Here we show that drinking H_2_ water may potentially offer a great advantage over other forms of antioxidant therapy, particularly for chronic pathological conditions such as PD.

## Results

### Hydrogen water made by bubbling H_2_ gas and using electrochemical reaction of magnesium

**Figure 1:**
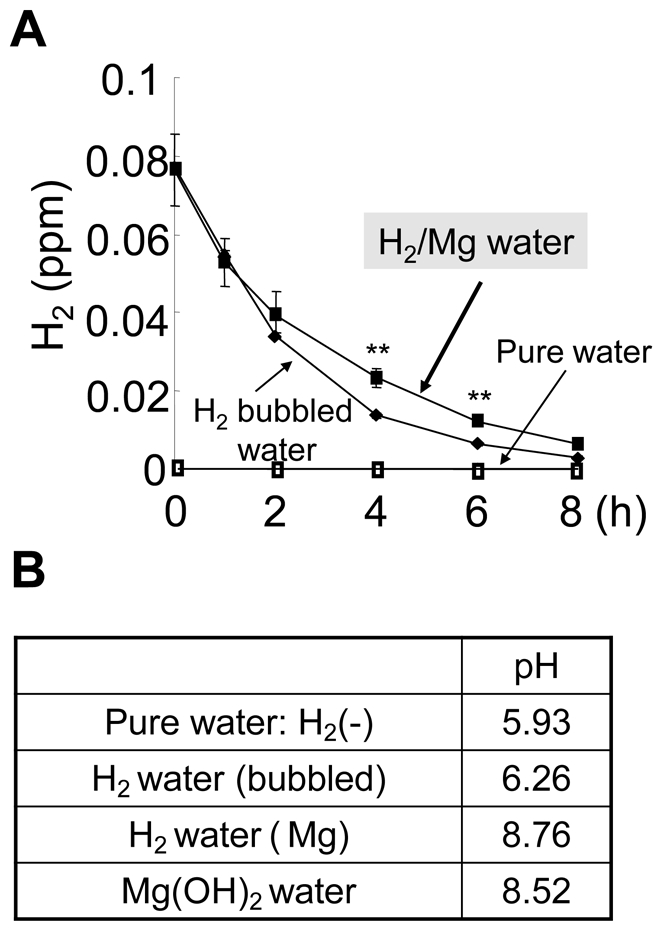
Time-dependent decrease of H_2_ content in each drinking water and their pH. Hydrogen water was made by directly bubbling H_2_ gas produced by electrolysis (H_2_ bubbled water) or chemical reaction using Mg (Mg/H_2_ water). **P<0.01 compared to H_2_ bubbled water. Error bars represent mean ± SEM.

Hydrogen water (H_2_ water) could be made by several methods. In the present experiments, H_2_ water made by two relatively easy and safe ways was tested. The H_2_ content in H_2_ water, made by either dissolving electrolyzed hydrogen into pure water (H_2_ bubbled water) or utilizing electrochemical reaction of magnesium with water (H_2_/Mg water), declined with a half-time of ∼2 h and almost disappeared after 8 h (Figure 1). The time course of H_2_ content was similar in H_2_ bubbled water and H_2_/Mg water except at 4 and 6 h, suggesting that H_2_ was better maintained in H_2_/Mg water, though the mechanism was not clear.

### The effect of H_2_ water on acute MPTP neurotoxicity

**Figure 2:**
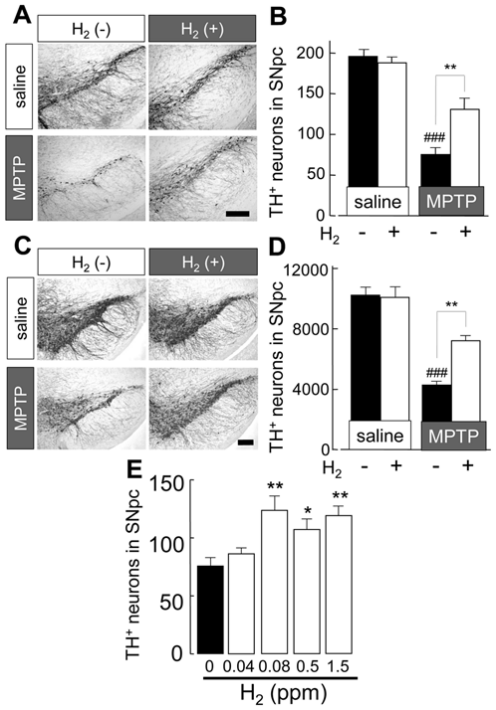
Drinking H_2_ bubbled water reduced loss of dopaminergic neurons in substantia nigra induced by acute injection of MPTP. (A) Representative photomicrographs illustrating tyrosine hydroxylase (TH)-staining in substantia nigra (SN) of indicated treatment animal groups. TH-staining from mice with saline injection drinking non-H_2_ water or H_2_-bubbled water and those from MPTP-injected mice drinking non-H_2_ water or H_2_ bubbled water. Scale; 200 µm. (B) Average number of TH-positive neurons in SN pars compacta (SNpc), measured in 20 µm coronal sections (n = 4–6). (C) Representative photomicrographs illustrating TH immunoreactivity in the SN of indicated treatment animal groups. Staining is more intense than in (A) because of increased section thickness (30 µm) required for stereological analysis. Scale; 200 µm. (D) Quantification of TH-positive neurons by stereology, as described in Materials and Methods. (n = 4 each) (E) TH-positive neurons in SN from mice treated with 4 different concentration of H_2_ (0.04, 0.08, 0.5, 1.5 ppm) in drinking water (n = 4–6). One-way ANOVA; ^###^ *P*<0.001 compared to saline with non-H_2_ water; *P<0.05, **P<0.01 compared to MPTP with non-H_2_ water. Error bars represent mean ± SEM.

To simply observe the effect of H_2_ in drinking water, H_2_-bubbled water was used for acute MPTP neurotoxicity tests. Mice were given H_2_ water or non-H_2_ water for 7 days prior to the MPTP administration and kept receiving it until the mice were killed and brains were extirpated. Since the H_2_ content disappeared within 8 h (Figure 1), water supply was restricted to 8 h per day so that most of the water was taken by the mice during the first few hours of each day. Systemic administration of MPTP caused a significant decrease in the number of dopaminergic neurons in substantia nigra pars compacta (SNpc) (38% of that after saline & non-H_2_ water) compared to those in saline injection group, as shown by the number of tyrosine hydroxylase (TH)-positive cells (Figure 2A). Dopaminergic fibers in the substantia nigra pars reticulosa (SNpr) were also apparently reduced. In mice treated with H_2_ water, the loss of dopaminergic neurons in SNpc was about a half of that in mice drinking non-H_2_ water (54% of saline & non-H_2_ water) and showed a significant reduction in the loss of neurons in SNpc (Figure 2B). In saline injected groups, there were no apparent effects of H_2_ on the number of dopaminergic neurons in SNpc (Figure 2A, 2B). This result was also supported by stereological analysis, a better method for unbiased cell counting (Figure 2C, 2D). The number of TH-positive neurons was significantly decreased by MPTP administration, to 40% of controls (4180±309, MPTP & non-H_2_ water; 10335±491, saline & non-H_2_ water). However, drinking H_2_ water significantly attenuated this decrease of neurons by MPTP without any decrease in saline injected mice (7105±325, MPTP & H_2_ water; 10094±716, saline & H_2_ water). Morphological observations also supported a lesser decrease of TH-immunoreactivity in both SNpc and SNpr. This rigorous stereological analysis fully supports the results obtained using conventional counting methods. Consequently, the protective effect of H_2_ was proved by two different counting methods. The effects of H_2_ water were dose-dependent, with a maximal effect at a much lower concentration (0.08 ppm) than saturated concentration of H_2_ (1.5 ppm) (Figure 2E).

### Mg/H_2_ water and its effect on acute MPTP model mice

**Figure 3:**
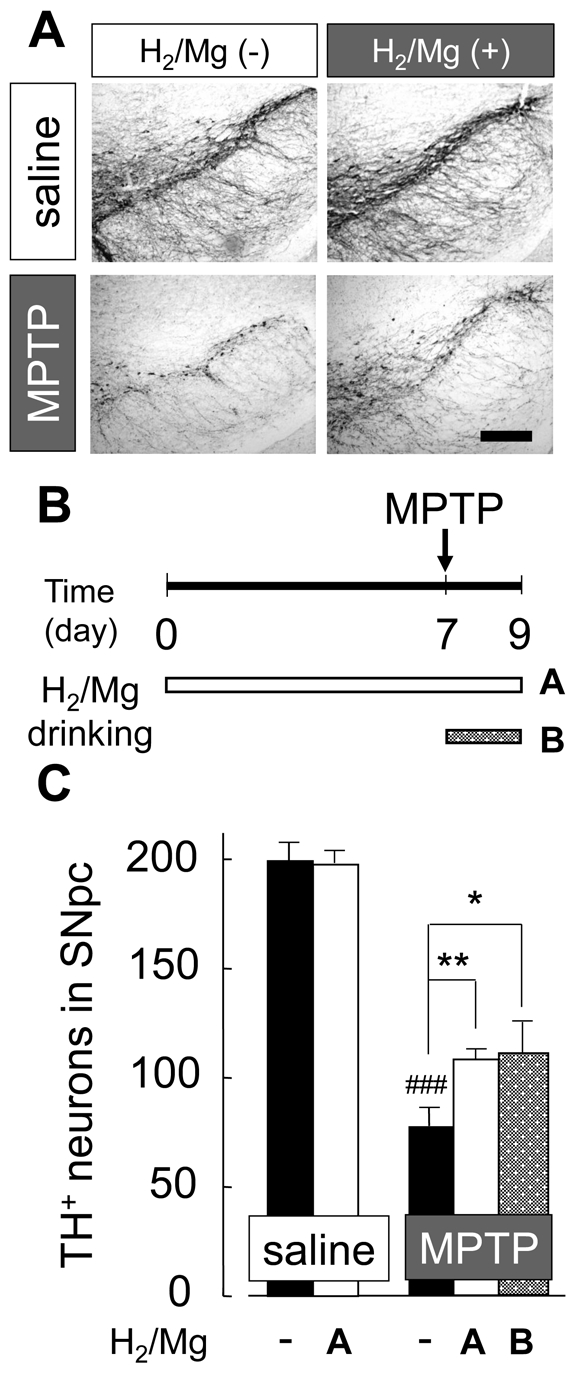
Drinking H_2_/Mg water before and after acute injection of MPTP reduced loss of dopaminergic neurons. (A) TH-positive dopaminergic neurons in SN. Mice were treated with non-H_2_ water or H_2_/Mg water before and after acute MPTP injection. Scale; 200 µm. (B) Schedule for drinking H_2_ water before (i) and after (ii) MPTP injection. (C) Average number of TH-positive neurons in mice with acute injection of saline or MPTP. One-way ANOVA; ^###^ *P*<0.001 compared to saline with non-H_2_ water; *P<0.05, **P<0.01 compared to MPTP with non-H_2_ water. Error bars represent mean ± SEM.

For further experiments, H_2_/Mg water was used, which was made by a much easier and safer procedure. H_2_/Mg water contained about 0.08 ppm H_2_. H_2_/Mg water showed a similar protective effect as H_2_-bubbled water on the loss of dopaminergic neurons in acute MPTP model mice. Without MPTP administration, H_2_/Mg water had no effect on the number of TH-positive cells (195±8; non-H_2_/Mg water, 194±4; H_2_/Mg water, Figure 3A, 3C). To test whether or not drinking H_2_/Mg water was effective even after suffering oxidative stress, we compared two different procedures (Figure 3B); one was giving H_2_/Mg water 7 days prior to the acute MPTP administration (Figure 3B; shown as i) and the other giving H_2_/Mg water only after MPTP administration (Figure 3B; shown as ii). The result showed that drinking H_2_/Mg water reduced the loss of dopaminergic neurons even after MPTP injection (Figure 3B, 3C; 16% & 17% recovery in drinking protocol shown as A&B, respectively). This may imply that drinking H_2_ water might be effective even after the onset of oxidative stress-induced PD.

**Figure 4:**
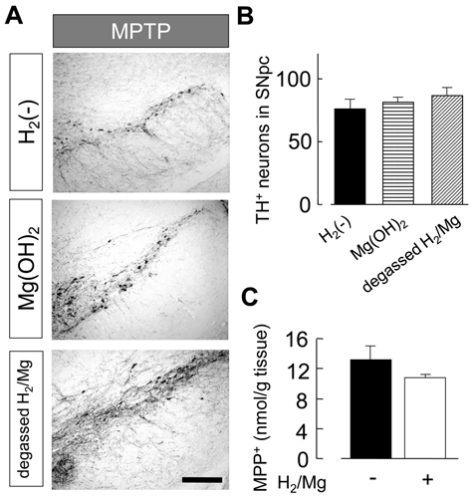
H_2_, but not Mg ion and alkalinity, protected the neuronal damage from acute MPTP neurotoxicity. (A) TH-positive neurons in SN in mice drinking pure water (non-H_2_ water), Mg(OH)_2_ water or degassed H_2_/Mg water. Neither Mg(OH)_2_ water nor degassed H_2_/Mg water had significant effect on MPTP neurotoxicity. (B) Average number of TH-positive neurons in SNpc in mice drinking non-H_2_ water, Mg(OH)_2_ water, and degassed H_2_/Mg water. (C) The amount of MPP+ in striatum in mice drinking H_2_/Mg water (white) and pure water (black) was not significantly different. MPP+ was measured 3 h after the last MPTP administration, when the concentration of MPTP reached maximum. One-way ANOVA. Error bars represent mean ± SEM.

Since H_2_/Mg water was alkaline (pH ∼8.8, Figure 1), we tested the effect of alkalinized water (up to pH 8.8) made by adding Mg(OH)_2_ to pure water. We also tested the effect of Mg and other metal elements, Al and Zn which might possibly exist in H_2_/Mg water, by leaving the H_2_/Mg water for 24 h (degassed H_2_/Mg water) so that all the H_2_ had gone. Neither of these solutions had any protective effect against MPTP-derived neurotoxicity (Figure 4A, 4B). Thus, the effect of the H_2_/Mg water was not due to metal elements or to its alkalinity but to its content of H_2_.

MPTP is converted into MPP+ which is indispensable for MPTP-derived neurotoxicity. We therefore measured the amount of MPP+ in the striatum where it was incorporated by DAT. The amount of MPP+ in the striatum was not significantly different in animals drinking H_2_ water and non-H_2_ water (Figure 4C). Therefore, the effect of H_2_ water against the loss of dopaminergic neurons in SNpc by MPTP was not related to the metabolism of MPTP.

### The effect of H_2_ water in chronic MPTP infusion model

**Figure 5:**
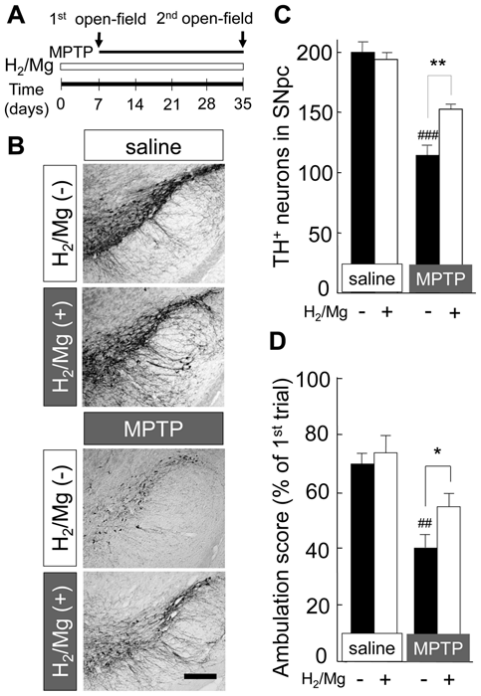
Drinking H_2_/Mg water attenuated neurotoxicity and behavioral phenotype induced by chronic infusion of MPTP. (A) Schedule for continuous infusion of MPTP and behavioral observation. Mice started to drink non-H_2_ water or H_2_/Mg water 1 week before infusion of minipump. The 1^st^ open-field test was performed in the morning of pump infusion. The 2^nd^ test was performed 28 days after pump infusion. (B) TH-staining from mice with saline-infusion drinking non-H_2_ water (I) or H_2_/Mg water (II) and those from mice with MPTP-infusion drinking non-H_2_ water (III) or H_2_/Mg water (IV). (C) Average number of TH-positive neurons in mice with saline- or MPTP-infusion (n = 6). Brain samples were obtained from six 20 µm coronal SN sections. (D) Suppression of open-field activity by chronic infusion of MPTP was partially recovered by drinking H_2_/Mg water. Relative ambulation score at the 2^nd^ measurement was expressed as percentage of the 1^st^ measurement (n = 6 for each group). One-way ANOVA; ^###^ *P*<0.001 compared to saline with non-H_2_ water; *P<0.05, **P<0.01 compared to MPTP with non-H_2_ water. Error bars represent mean ± SEM.

Despite the lack of parkinsonian symptoms in the acute MPTP model in rodents, probably due to the low level of MAO-B in the rodent brain's capillaries [15], behavioral impairment could be observed in the chronic MPTP infusion model using an osmotic minipump to deliver the MPTP for long period [16]. This could provide a better model for human PD. In this experiment, mice were supplied with H_2_ water or non-H_2_ water 7 days prior to the pump implantation and the water supply continued until brain extirpation 28 days later (Figure 5A). Chronic infusion of MPTP induced a loss of TH-positive dopaminergic neuron in SNpc (Figure 5B). In mice drinking H_2_ water, the loss of TH-positive cells (76% of control: saline & non-H_2_ water) was less than that in mice drinking non-H_2_ water (56% of control: saline & non-H_2_ water) (Figure 5C). For the behavioral test, the ambulation scores in the open-field test were compared. Using the slightly modified open-field test reported previously [16], the ambulation score was expressed as percentage of that obtained in the first trial. Drinking H_2_ water did not show any significant change in the ambulation score in mice with saline infusion (69±3% with non-H_2_ water and 73±6% with H_2_ water, respectively). The ambulation score in mice with chronic MPTP infusion with non-H_2_ water was 40±4%, while in mice with H_2_ water was 54±4% (Figure 5D). Other behavioral tests, for example the rotarod test and tail suspension test, were also examined but no significant effects were observed following chronic MPTP infusion (data not shown).

### Hydrogen water reduced 4-HNE production in SN dopaminergic neuron

**Figure 6:**
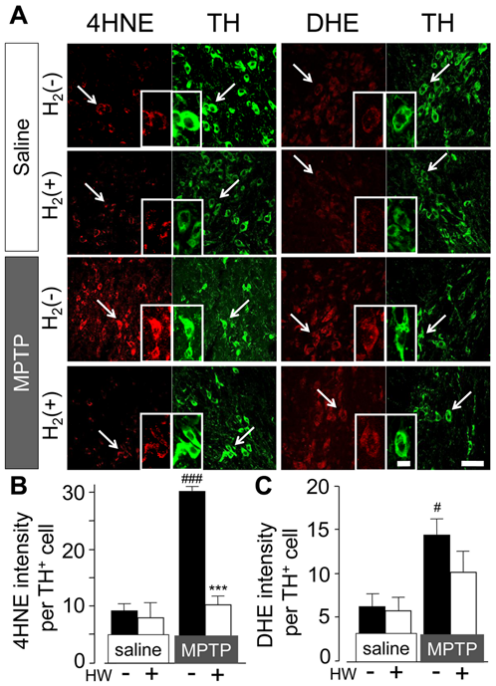
H_2_ water reduced 4-HNE, but not DHE fluorescence in SN after MPTP administration. (A) Fluorescent images of 4-HNE, DHE, and TH in SN (n = 3∼4). Mice treated with non-H_2_ water or H_2_/Mg water with injection of saline or MPTP. All samples were acquired 24 h after administration of saline or MPTP. Scale: 50 µm. (B, C) Quantification of 4-HNE (B) and DHE (C) intensity in TH-positive cells in SN. One-way ANOVA; ^#^ *P*<0.05, ^###^ *P*<0.001 compared to saline with non-H_2_ water; ^***^ *P*<0.001 compared to MPTP with non-H_2_ water. Error bars represent mean ± SEM.

4-HNE is an aldehyde toxic end product of lipid peroxidation and one of the markers of membrane lipid peroxidation induced by cytotoxic radicals such as •OH [17]. Also, 4-HNE is reported to mediate the induction of neuronal apoptosis in the presence of oxidative stress [18]. In the acute MPTP model, protein level of 4-HNE in midbrain is increased [12], [19]. In saline-injected mice, 4-HNE immunoreactive fluorescence in TH-positive neuron was minimal (Figure 6A, 6B). In contrast, in MPTP-treated mice, 4-HNE fluorescence was increased significantly 24 h after the last injection of MPTP.

In mice drinking hydrogen water, the baseline level of 4-HNE fluorescence was unchanged, but the striking increase in fluorescence following MPTP administration was virtually annulled, to a level that was not significantly above baseline (Figure 6B).

### Hydrogen water failed to reduce the production of intracellular superoxide in SN dopaminergic neuron

Intracellular superoxide (O_2_•^−^) was detected by administration of O_2_•^−^ indicator, dihydroethidium (DHE) [20]. When DHE is oxidized by O_2_•^−^, it binds to DNA and a bright red fluorescence is observed within the cell body.

The DHE intensity in TH-positive cell was significantly increased in the acute MPTP-treated mice compared to saline-treated mice (Figure 6A, 6B). Mice drinking hydrogen water appeared to show a slight decrease of DHE fluorescent intensity compared to non-hydrogen water mice with MPTP-injection, but this was not statistically significant (*p* = 0.21). As for microglia, which is one of the sources of superoxide release in SN, microglia did not show their activation 24 h, but 48 h after MPTP treatment. In mice drinking H_2_ water, the morphological change of microglia was also observed 48 h, but not 24 h after MPTP administration (Figure S1).

### Accumulation of cellular 8-oxoguanine in striatum

**Figure 7:**
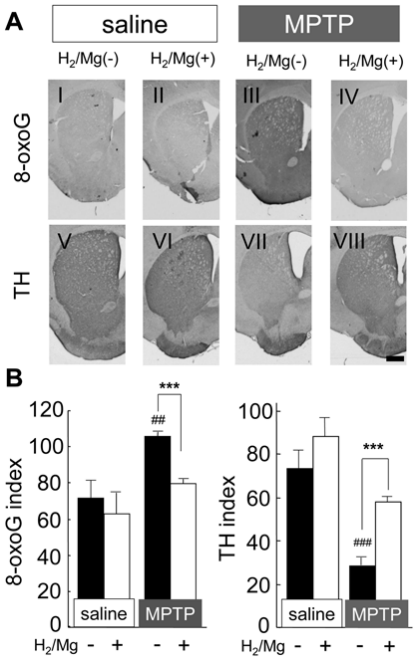
H_2_ water decreased the accumulation of 8-oxoG in striatum after MPTP administration. (A) Acute administration of MPTP significantly increased the accumulation of mitochondrial 8-oxoG (I and III) and induced the loss of TH-positive neurons and fibers (V and VII). However, in mice drinking H_2_/Mg water, no significant 8-oxoG accumulation (II and VI) nor TH-positive staining (VI and VIII) was observed. Brains were extirpated 24 h after administration of saline or MPTP. Scale: 500 µm. (B) Immunoreactive index for 8-oxoG and TH (n = 4 each). One-way ANOVA; ^##^ *P*<0.01 and ^###^ *P*<0.001 compared to saline with non-H_2_ water; ***P<0.001, compared to MPTP with non-H_2_ water. Error bars represent mean ± SEM.

MPTP-induced loss of dopaminergic neuron is associated with the accumulation of 8-oxoguanine (8-oxoG) in the nigro-striatal pathway [5]. 8-oxoG is the major form of guanine oxidized by •OH, and is accumulated in both mitochondrial and nuclear DNA [21]. Hence, 8-oxoG is widely used as an index of DNA oxidative stress. The distinction between 8-oxoG accumulation in mitochondrial and nuclear DNA can be made using immunohistochemical techniques [5], [22]. In our study, we investigated the accumulation of mitochondrial 8-oxoG in the striatum, where the dopaminergic nerve terminals end. Mitochondrial 8-oxoG showed significant accumulation in the striatum 24 h after acute MPTP administration. H_2_ water reduced this accumulation to a level not significantly different from the control (Figure 7A, 7B). At 24 h after acute MPTP administration, microglia in the striatum were activated in mice drinking both H_2_ and non-H_2_ water (data not shown), and similarly in the SN 48 h after MPTP administration (Figure S1).

## Discussion

This study shows that drinking water containing H_2_ (H_2_ water) attenuated the acute neurotoxic effects of MPTP on dopaminergic neurons. H_2_ water was also effective on the behavioral impairment produced by chronic administration of MPTP, the major symptom of Parkinson's disease (PD). We found that low concentration of H_2_ in drinking water, well below saturating concentrations, showed neuroprotective effects against the MPTP-induced loss of dopaminergic neurons and neuronal fibers in nigro-striatal pathway, and that this could be seen even after the start of the neurotoxic insult. H_2_ water also reduced the amount of ROS-derived oxidative products such as 4-HNE and 8-oxoG, which would be primary causes of neuronal apoptosis in dopaminergic neurons. Thus, our study may pave the way toward a new neuroprotective strategy using H_2_ water in PD patients.

PD is a progressive, degenerative disorder of the nervous system, meaning that it becomes increasingly disabling over time. It is a chronic, life-long condition. Various causes have been speculated: dopaminergic neurons can die or become damaged by infection, trauma, or toxins found in the environment, like MPTP. There may be also some genetic links. The impairments by all of these causes are generally related to varying degrees of oxidative stress [23]. Oxidative stress contributes to the cascade leading to dopamine cell degeneration in PD. Studies revealed that several biomarkers of oxidative damage are elevated in SN of PD brain [9]. Also, dopaminergic neurons may be a particularly fertile environment for the generation of ROS, as the metabolism of dopamine produces hydrogen peroxide and superoxide radicals, and auto-oxidation of dopamine produces dopamine-quinone, a molecule that damages proteins by reacting with cysteine residues [9], [24].

Taking these reports into consideration, reducing oxidative stress or oxidative damage would become a potential way to prevent PD. H_2_ acts as a therapeutic antioxidant by selectively reducing cytotoxic oxygen radicals [1]–[4], [14], [25], [26], either by inhalation or consumption. Although inhalation of H_2_ may act more rapidly, it is not practical in daily life or suitable for continuous consumption. H_2_ water is clearly more convenient, and fits well within a normal life-style. The most convenient form of H_2_ water is H_2_/Mg water. In our experiments, neither the alkalinity nor the magnesium had any effect but the small amounts of dissolved H_2_ turned out to have significant neuroprotective and anti-oxidant effects.

As for the concentration of H_2_, it was reported that H_2_ dissolved in arterial blood was increased by the inhalation of H_2_ in proportion to the concentration inhaled, from 0 to 4%, together with O_2_ and N_2_O; the amount of H_2_ dissolved in venous blood was less than that in artery blood, suggesting that H_2_ had been incorporated into tissues [1]. Recently it was also reported that the blood concentration of H_2_ which was incorporated from the stomach using water with saturated H_2_ was 5 µM [3]. We tried to measure the concentration of H_2_ in rat striatum with cyclic voltammetry analysis using a H_2_ electrode (Teflon-coated platinum electrode for the cerebral blood flow study, 200 µm in diameter) [27] inserted through a previously-mounted guide-cannula [28] (See Materials and Methods S2). We could detect the change in H_2_ in the striatum during the inhalation of H_2_ (Figure S2). It increased immediately when the rats started to inhale the H_2_ gas and reached a plateau within 10 minutes (0.22 ppm; 110 µM), but decreased soon after stopping inhalation (t_1/2_ = 343 s; n = 3). However, we could not detect any change in H_2_ concentration on instilling saturated H_2_ water into the stomach in anesthetized rats or in free-moving rats drinking H_2_-water. If there is 160 fold decrease in H_2_ concentration between stomach and blood [3], there would be an even greater decrease between the H_2_ concentration in drinking water (maximum 1.5 ppm) and that in striatal tissue. This suggests that the tissue H_2_ concentration would be below detection level. Therefore, even though tissue H_2_ would be expected to rise after drinking H_2_ water, currently there is a technical problem in detecting such low levels.

In the PD model, ROS can be released from both cytosol and mitochondria of DA neuron [29]–[32]. Administration of MPTP causes its dysfunction via inhibition of complex I, which is associated with the electron transport chain [11]. Subsequent studies have identified abnormalities in complex I activity in PD [9], [33]. Mitochondrial dysfunction causes massive production of ROS, especially O_2_•^−^. At the nerve terminal of dopaminergic neuron in the striatum, where there are abundant mitochondria, more production of O_2_•^−^ is predicted [9]. Furthermore, O_2_•^−^ transforms into •OH by several steps including the Fenton reaction, which is catalyzed by ferrous iron (Fe^2+^). Fe^2+^ can react with hydrogen peroxide (H_2_O_2_) (produced during oxidative deamination of dopamine) to generate •OH that can damage proteins, nucleic acids, and membrane phospholipids, leading to cellular degeneration [34]–[36]. We showed that a significant increase in markers characteristic of oxidative damage in the nigro-striatal pathway, such as 4-HNE, mitochondrial 8-oxoG, and DHE, occurred 24 h after MPTP administration. 4-HNE and 8-oxoG are well-known markers for oxidative insult mainly caused by •OH. 4-HNE was observed in membrane surface of TH-positive dopaminergic neuron in SN, and mitochondrial 8-oxoG was accumulated in the striatum over the same period (24 h after MPTP administration). It has been previously reported that mitochondrial 8-oxoG, the •OH -oxidative form of guanine and a marker of DNA damage, is accumulated the striatum of MPTP-injected mice [5]. However, hydrogen water significantly decreased the amount of 4-HNE in the SN and mitochondrial 8-oxoG in the striatum. This indicates that hydrogen acted by reducing the primary production of •OH in the nigro-striatal pathway.

A DHE fluorescence signal is detected when DHE and O_2_•^−^ react and the oxidized DHE is incorporated into DNA. So, DHE is widely used for imaging intracellular O_2_•^−^ both *in vitro* and *in vivo* [37]–[39]. During MPTP neurotoxicity, O_2_•^−^ is released from not only neuronal mitochondria, but also by auto-oxidation of dopamine in dopaminergic neuron [40]. Also, NADPH oxidase in activated microglia is a source of O_2_•^−^ in the nigro-striatal pathway [41]. However, in our experiments, we observed the production of O_2_•^−^ at 24 h, before microglial activation. Although microglia are key players in the release of O_2_•^−^, microglial NADPH activity is enhanced together with microglial activation and the peak of NADPH activity is only observed 48 h after administration of MPTP [42]. In our case, microglia was activated following the degeneration of dopaminergic neurons, not before, as shown in Figure S1. Although some microglia in the SN were activated in 24 h, we can conclude that DHE signals which we observed in 24 h after MPTP injection mostly reflected neuronal O_2_•^−^ production in SN.

In a previous study, hydrogen reduced •OH but not other kinds of reactive oxygen/nitrogen species [1]. However, *in vivo*, there is little knowledge whether hydrogen shows selective reduction of ROS [4]. In our experiment, DHE intensity was not significantly reduced by hydrogen water in MPTP-treated mice, indicating that hydrogen water showed less or no reduction of O_2_•^−^. This accords with the previous report that hydrogen showed a selective reduction of ROS [1]. On the othet hand, another study has shown that hydrogen water can reduce the amount of O_2_•^−^ *in vivo* [4]. These opposite results may relate to the different concentrations of hydrogen in drinking water. Our hydrogen water contained a much lower amount of hydrogen than saturated hydrogen water, which may not be enough to reduce O_2_•^−^.

We provided chronic MPTP administration model using osmotic minipump modifying the previous method. However, the number of TH-positive neurons in chronic MPTP model mice was more than acute MPTP model mice. Continuous MPTP infusion model caused less acute toxicity because the peak concentrations of MPP+ in striatum were lower [16]. Several reports indicated that the loss of dendritic processes in striatum and nigral TH-positive neurons' cell bodies were not permanent and recovery of striatal and nigral TH-immunoreactivity were observed after MPTP is eliminated systemically [42]–[44]. Such increases in TH-immunoreactivity may reflect sprouting of residual fibers [44] or the *de novo* appearance of TH-positive neurons in dopamine-depleted striatum [45]–[47], but more likely represent a compensatory mechanism for chronically reduced dopamine levels as suggested from post-mortem studies of PD brains [48]. Taking into consideration of these reports, we may conclude that the number of TH-positive neurons in chronic MPTP model does not decrease as much as in acute MPTP model because chronic recovery and damage of TH fiber occurred simultaneously in nigro-striatal pathway. Nevertheless, chronic administration induced behavioral impairment. This impairment may be due to degeneration of dopaminergic and noradrenergic neurons, i.e. loss of dopamine and norepinephrine, but not serotonin, from the target areas of the respective neurons. And what is more, chronic neurotoxicity induced formation of neuronal inclusion bodies and degeneration of catecholaminergic neurons, similar to the effects of chronic administration of rotenone which is also a mitochondrial complex I inhibitor [49]–[51]. Therefore, continuous mitochondrial inhibition may impair the ubiquitin proteasome system, which in turn recreates a disease state that mimics human PD better than acute mitochondrial inhibition.

Dopaminergic lesions induced by MPTP are commonly used to model of PD, and although MPTP effectively mimics the dopaminergic neuropathology of PD in mice, it fails to produce PD-like motor deficits [52]. Although continuous MPTP administration with an osmotic minipump mimics many features of the human disease [16], no significant effects in behavioral impairment except only open-field test showed significant change but not in other test such as rotarod test, tail suspension test, and ring test (data not shown). In acute PD model mice, age-related severity of dopaminergic neurodegeneration and motor dysfunction to MPTP neurotoxicity was reported. Although young (10 weeks) mice showed no mortality in either MPTP treatment, older (14–15 months) mice exhibited mortality from only two injections of MPTP during the experimental period [53]. Though the effects of H_2_ water on behavioral response were not tested in older mice in the current study, our results raised a possibility that H_2_ water may reduce the movement disorder in PD patients.

H_2_ would be also useful for other diseases which are caused by oxidative stress. Already, H_2_ has been found effective for not only ischemic injury but also hepatic damage and cognitive disease [1]–[3], [23]. On the other hands, 8-oxoG is accumulated both in nuclear and mitochondrial genomes during aging [54], and dramatic increase in 8-oxoG accumulation was reported in patients with tumors [55] and other kinds of neurodegenerative disease such as PD [56], Alzheimer's disease [57] and amyotrophic lateral sclerosis [58]. Several experiments using genetic knockout mice revealed that the correlation between 8-oxoG accumulation and functional disorder of repair enzymes such as MTH1 [59], MUTYH [60], and OGG1 [61], suggesting that H_2_ may also suppress tumorigenesis.

H_2_ gas itself has a risk of flammability and explosion. However, drinking H_2_ water would be much safer and easier, being more practical. In addition, the required concentration of H_2_ in drinking water is not necessarily high. Our study also revealed that drinking H_2_ water even after acute MPTP administration was effective as well. While there is no known way to prevent PD, the current studies strongly suggest that drinking H_2_ water could reduce the risk of life style-related oxidative stress and related neurodegenerative diseases.

## Materials and Methods

### Hydrogen-containing water and its H_2_ content

Hydrogen-containing water (H_2_ water) was made by two ways. One way was by dissolving H_2_ gas, produced by electrolysis of water (hand-made by Panasonic Electric Works Co., Ltd., Japan), directly into pure water. Another way was utilizing electrochemical reaction between magnesium and water (H_2_O): Mg+2H_2_O→ Mg^2+^+2OH^−^+2H_2_. A magnesium stick composed of 90% Mg, 9% alminium and 1% zinc (6 mm diameter, 10 cm length, Nakagawa Metal, Japan) was surface-cleaned with 0.1 N acetic acid and dipped into pure water for about 1 minute. The maximum H_2_ content with this method was about 0.08 ppm, measured with a hydrogen electrode (DH-35A, TOA DKK Co. Ltd., Japan). Electrolyzed H_2_ water was also adjusted to have 0.08 ppm H_2_ unless otherwise indicated.

### pH of H_2_ water and Mg(OH)_2_ solution

The pH of H_2_ water made by the chemical reaction with Mg was about 8.8 due to the formation of Mg(OH)_2_, while the pH of pure water was 5.9. To investigate the effect of the alkaline solution (pH 8.8), Mg(OH)_2_ (∼80 µM) was added to pure water.

### Treatment of mice with H_2_ water

Animal protocols were approved by the Animal Care and Use Committee of Kyushu University. Male C57BL/6J (CLEA Japan Inc., Japan) mice of 8∼12 weeks old were maintained on a 12∶12 h light/dark cycle. H_2_ water was made freshly each day and 40 ml was delivered in glass bottles with tight rubber caps per 4 mice for 8 h (4–12 p.m.). Unless otherwise indicated, mice were treated with H_2_ or non-H_2_ water for 7 days prior to MPTP administration. For MPTP chronic model, treatment with H_2_ or non-H_2_ water continued after MPTP administration.

### Acute injection and continuous infusion of MPTP

For acute injection of MPTP, MPTP-HCl (20 mg/kg free base, SIGMA, USA) was administrated to mice intraperitoneally (i.p.) three times (2 h apart) and brains were extirpated under anesthesia 48 h after the last MPTP injection. MPTP was dissolved in 0.9% NaCl. For the continuous infusion of MPTP, an osmotic minipump (ALZET model 2004, USA) was transplanted subcutaneously, releasing either saline solution or MPTP solution at 45 mg/kg daily. After implantation, the incision was sutured, and disinfected with 70% ethanol every day. Twenty-eight days after the start of MPTP infusions, brains were extirpated under anesthesia. We checked that no solution was retained in the pump after the brain was removed.

### Immunohistochemistry of tyrosine hydroxylase (TH)

TH-positive neurons were stained as described previously [5]. Brain sections (20 and 40 µm thick in SN and striatum, respectively) were incubated in blocking solution (Block Ace, Dainippon Pharmaceutical, Japan) for 30 min at room temperature, and then incubated with primary antibody (rabbit anti-TH antibody; 1∶500, AB152, Chemicon, USA) in 10% Block Ace in PBS, at 4°C overnight. The rinsed sections were immersed in a solution of 3% H_2_O_2_ in methanol/PBS (1∶1) for 30 min at room temperature, and then processed by the Vectastain ABC kit (Vector, USA) with secondary antibody (biotinylated anti-rabbit IgG, 1∶200, Vector, USA). The peroxidase reaction product was detected using 3′3′-diaminobenzidine-tetrahydrochloride (DAB, Vector, USA). All sections were then washed in PBS, cover-slipped, and analyzed by AxioSkop2 equipped with with a CCD camera, AxioCam (Carl Zeiss, Germany). The number of dopamine neurons in the SNpc was estimated by counting all TH-positive neurons of two hemispheres from six coronal sections (20 µm thick) per animal that were distributed every 100 µm along the rostral–caudate axis of the SN (−3.08 to −3.64 mm caudal to bregma) [62].

Microglial cells were stained as described in Materials and Methods S1 [References S1].

### Stereological analysis of nigral Dopaminergic neuron

We performed stereological analysis using slightly modified method as described previously [63]–[65]. Coronal sections (30 µm thickness) were obtained through SNpc (−2.70 mm to −3.80 mm relative to bregma) on MICROM cryostat. Free-floating sections were incubated by Block Ace (Dainippon Pharmaceutics, Japan) for 30 min followed by incubation of primary antibody (anti-TH antibody, Chemicon, USA, 1∶3000 in 10% Block Ace) for 2 days at 4°C. After the rinsed sections were immersed in a solution of 3% H_2_O_2_ in methanol/PBS (1: 1) for 10 min at room temperature, secondary antibody (biotinylated goat anti-rabbit IgG, 1∶400, Vector, USA) were incubated for 2 h, and then were processed by Vectastain ABC kit (Vector, USA). The peroxidase reaction product was detected using 3′3′-diaminobenzidinetetrahydrochloride (DAB, Vector, USA). All sections were coverslipped with Glycergel Mounting Medium (Dako, Denmark).

For stereological counts, Stereo Investigator analysis software (Stereo Investigator 8, MicroBrightField Inc., USA) was used to perform unbiased stereological counts of TH-immunoreactive cell bodies in the SNpc using optical fractionator method [66]. The boundary of SNpc was outlined under magnification of the 10x objective. Every third section (eight sections per brain) were obtained, and cells were counted with a 40x objective on a Nikon ECLIPSE 80i using a grid of 70×70 µm on a counting grid (75×100×12 µm) with 2 µm upper and lower guard zones. The absolute number of TH-positive neurons was directly calculated, and the Gundersen's coefficient of error in all samples were <0.07.

### Labeling of intracellular superoxide *in vivo*

Intracellular superoxide levels were detected by quantification of fluorescence of the oxidation product of dihydroethidium (DHE, Molecular Probes, USA). Oxidation of DHE by superoxide converts DHE, which exhibits weak blue fluorescence, to an ethidium derivative (oxy-Et) that exhibits peak fluorescence in the rhodamine spectrum (excitation 480 nm, emission 567–586 nm) [20]. DHE, which is cell permeant, enters the cell and, after oxidation, binds to DNA with a small shift in its emission spectrum to 567 nm. DHE has increased specificity for superoxide compared with other dyes which are more general reactive oxygen species probes. DHE solution (200 µl; 1 mg/ml in PBS) was administrated intravenously 5 h before extirpating brain. Brains were sliced into 40 µm thicknesses using a cryostat and free-floating sections were obtained from the part of SN, as mentioned above in TH immunohistochemistry.

For immunofluorescent staining of TH, sections were incubated with Block Ace for 30 min at room temperature. The brain sections were incubated overnight at 4°C with primary antibody, rabbit anti-TH antibody (chemicon, 1∶1000), and incubated for 4 h with Alexa fluor 488 goat anti-rabbit IgG (Molecular Probes, 1∶500, USA) at room temperature. Every treatment was followed by washing three times for 5 min with PBS. Sections were mounted in the permafluor aqueous mounting medium (Thermo, Japan) and were analyzed with a confocal laser scanning microscope (LSM510META, Carl Zeiss, Germany).

### Fluorescent labeling of 4-hydroxynonenal in SNpc

For detection of cellular lipid peroxide, we stained 4-hydroxynonenal (4-HNE). Briefly, free-floating sections were obtained from the part of SN, and incubated with primary antibody overnight following to the incubation with Block Ace. Then, sections were incubated with biotinylated anti-mouse IgG (Jackson, USA, 1∶400), followed by streptavidin Alexa 594 (Molecular Probes, USA, 1∶1000). After staining of 4-HNE, TH was stained as described above.

### Quantitative analysis of DHE and 4-HNE fluorescent signal

All samples were analyzed with a confocal laser scanning microscope (LSM510META, Carl Zeiss, Germany). For each slice 10 series of z-stack images corresponding to 2 µm on the z-axis were obtained and projected using LSM image browser (Carl Zeiss, Germany). For quantification of fluorescent intensity of DHE and 4-HNE, all images were converted to grayscale, and the average of pixel intensity of each cell was measured using Adobe Photoshop CS3 (Adobe Systems). Over 60 cells which were merged with TH immunofluorescence were observed per animal and mean value of intensity/cell/animal was calculated in each group.

### Immunohistochemistry of 8-oxoguanine in striatum

The method of staining 8-oxoG in mitochondrial DNA was described previously [5], [22]. To eliminate cellular RNA, the sections were incubated in 10 mM Tris-HCl (pH 7.5), 15 mM NaCl containing DNase-free RNase (5 mg/ml of heat-incubated RNase A, Sigma, USA) for 60 min at 37°C. In this condition, nuclear DNA is not denatured at all, therefore the anti-8-oxoG antibody cannot access to 8-oxoG residues buried in the intact chromatin in nuclei, while the antibody easily accesses to 8-oxoG residues in mitochondrial DNA which does not have a tight chromatin structure. To detect 8-oxoG in nuclear DNA, section has to be subjected to pre-treatment with 2N HCl for 1 h at room temperature, which allows an efficient denaturation of nuclear chromatin but an extensive degradation of mitochondrial DNA [22]. Free-floating sections pre-treated were incubated in Block Ace, for 30 min at room temperature, and then were incubated with primary antibody (N45.1 mAb 1∶100, Japan Institute for the Control of Aging, Japan) in 10% Block Ace, at 4°C overnight. The rinsed sections were immersed in a solution of 3% H_2_O_2_ in methanol/PBS (1: 1) for 15 min at room temperature, and then were processed by Vectastain ABC kit with a biotinylated secondary antibody, and the peroxidase reaction product was detected using DAB. Digital images were acquired using Axioskop2 plus equipped with a CCD camera, AxioCam. All sections from each experimental animal and group to be compared were processed in parallel.

### Quantitative morphometric analysis of 8-oxoG

All acquired digital images were processed uniformly, as described previously [5], at a threshold in a gray scale mode to subtract any background corresponding to the area without tissue, and the optical density (OD) of each immunoreactive area was calculated by Adobe Photoshop version 7.0 (Adobe Systems). From each individual animal, five representative sections were measured and the mean OD was calculated as an immunoreactivity index for each animals. All quantitative analyses were performed by an individual unaware of the experimental treatments.

### Open-field test for continuous MPTP infusion model mice

Before and 28 days after osmotic pumps were implanted, open-field tests were performed between 9 and 12 AM. A round open field with 60 cm in base diameter and 90 cm in upper diameter, surrounded by a 50 cm-high wall was used. During observation, a 100 W lamp-light was placed 80 cm above the field. Each mouse was put at the center of the field and the movements were recorded. Each mouse was tested for 3 min and the number of times they crossed a line drawn in the field was counted (modified from the method described previously [67]).

### MPP+ measurement

HPLC-UV detection (wavelength, 295 nm) was used to measure 1-methyl-4-phenylpyridinium (MPP*+*, SIGMA, USA) levels in striatum using the method as described previously [68], [69]. Mouse brains were subjected to be measured 1.5, 3, 6 h after MPTP was treated (n = 4). The standard solution of MPP+ (500 nM) was made by dilution of MPP+ stock solution (1 mM in 20% glycerol, 2.5% MeOH in pure water, pH 4.0 with H_3_PO_4_).

### Statistical Analysis

All data represent mean±standard error of means (SEM). The data were compared with one-way ANOVA, followed by Bonferroni test. Values of *p *<0.05 were considered statistically significant.

## Supporting Information

Figure S1 Immunohistochemistry of microglia in substantia nigra. Mice drinking non-H_2_ water (H_2_(−)) or H_2_ water (H_2_(+)) were treated with saline or MPTP. Brains were obtained 24 h or 48 h after the last injection of saline or MPTP. Microglial cells were immunostained with anti-Iba1 antibody (1∶1000, WAKO). Scale: 50 µm.
(3.97 MB TIF)

Figure S2 Representative concentration curve of H_2_ in striatum. Anesthetized rats were inserted H_2_ electrode to right striatum and currents were recorded by voltammetry. Rats started to inhale H_2_ gas at 200 s after the start of recording, and stopped at 800 s.
(0.39 MB TIF)

Materials and Methods S1 (0.02 MB DOC)

Materials and Methods S2 (0.02 MB DOC)

References S1 (0.02 MB DOC)
