# Supplementary figures and images for: Hydrogen in Drinking Water Reduces Dopaminergic Neuronal Loss in the 1-methyl-4-phenyl-1,2,3,6-tetrahydropyridine Mouse Model of Parkinson's Disease
Source: PLoS One. 2009 Sep 30;4(9):e7247. doi: 10.1371/journal.pone.0007247 (PMC2747267; doi:10.1371/journal.pone.0007247)

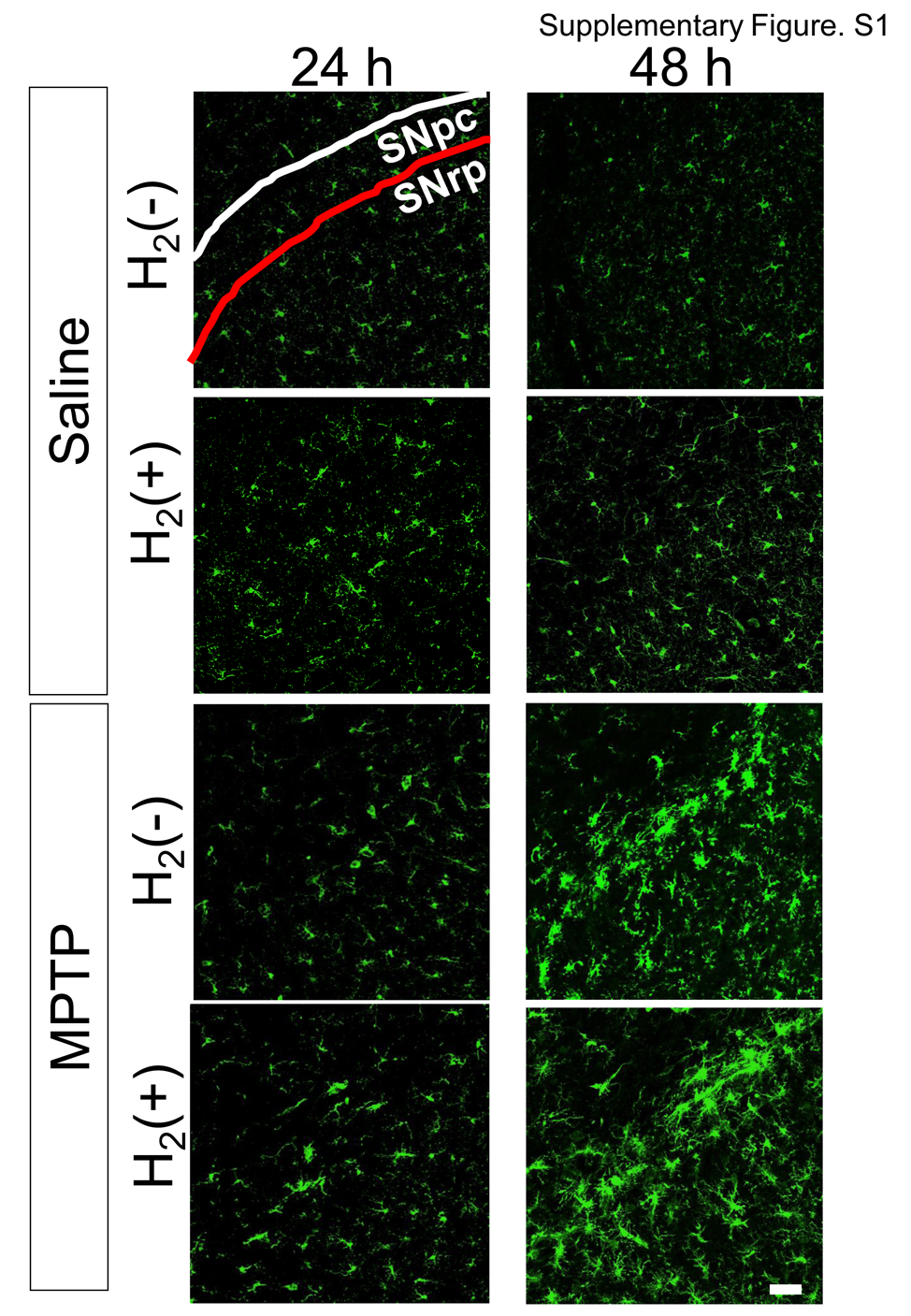

Supplement: Figure S1 — Immunohistochemistry of microglia in substantia nigra. Mice drinking non-H2 water (H2(−)) or H2 water (H2(+)) were treated with saline or MPTP. Brains were obtained 24 h or 48 h after the last injection of saline or MPTP. Microglial cells were immunostained with anti-Iba1 antibody (1∶1000, WAKO). Scale: 50 µm. (3.97 MB TIF) [file pone.0007247.s001.tif]

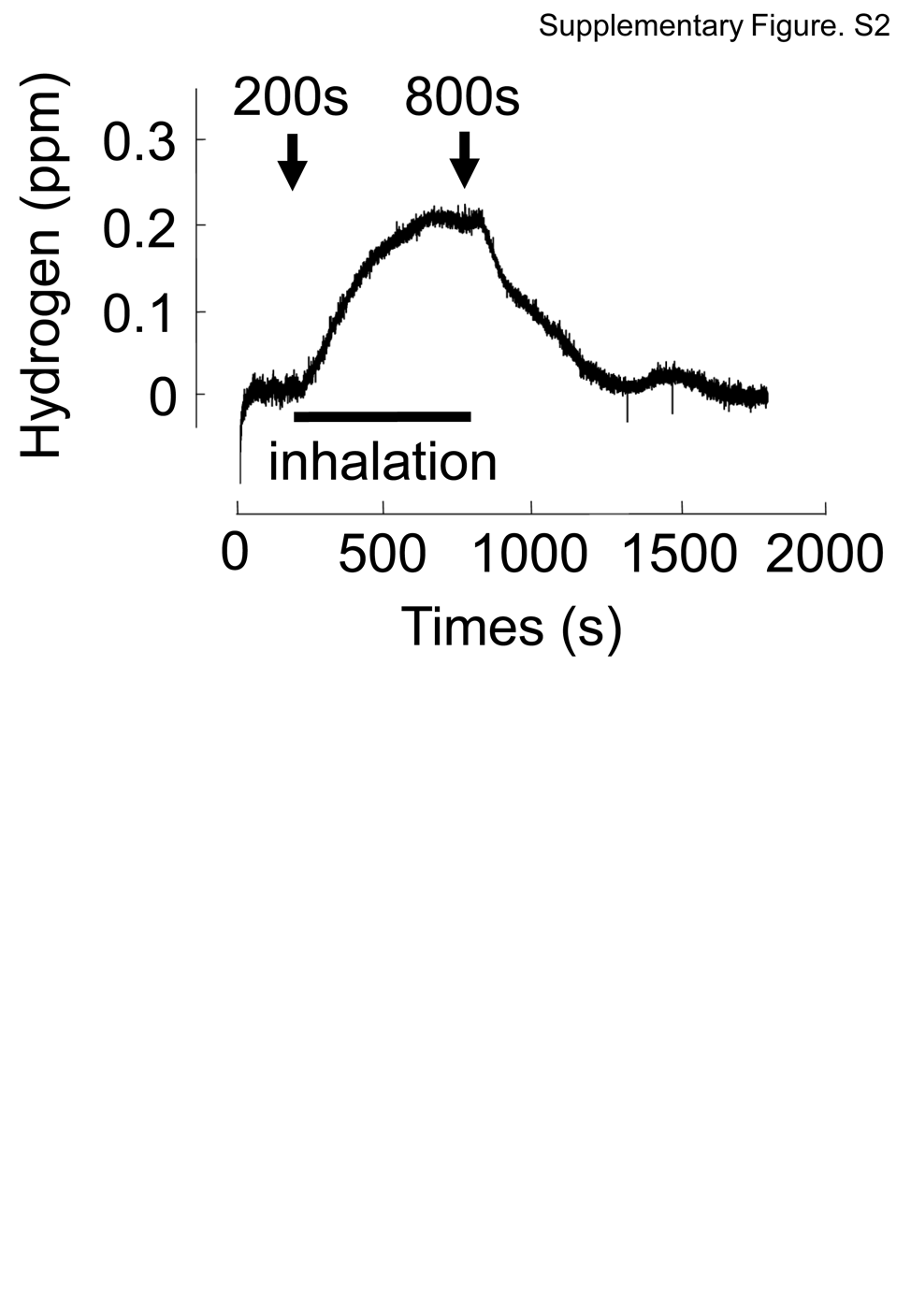

Supplement: Figure S2 — Representative concentration curve of H2 in striatum. Anesthetized rats were inserted H2 electrode to right striatum and currents were recorded by voltammetry. Rats started to inhale H2 gas at 200 s after the start of recording, and stopped at 800 s. (0.39 MB TIF) [file pone.0007247.s002.tif]
