# Supplementary material for: Hydrogen in Drinking Water Reduces Dopaminergic Neuronal Loss in the 1-methyl-4-phenyl-1,2,3,6-tetrahydropyridine Mouse Model of Parkinson's Disease
Source: PLoS One. 2009 Sep 30;4(9):e7247. doi: 10.1371/journal.pone.0007247 (PMC2747267; doi:10.1371/journal.pone.0007247)
Supplement: Materials and Methods S1 — (0.02 MB DOC) [file pone.0007247.s003.doc]

**Immunohistochemistry of microglia in SN**

Microglial cells were stained by slightly modified method described previously [S1]. Free-floating sections (40 μm) were incubated with Block Ace for 30 min, followed by application of primary antibody (rabbit anti-Iba1 antibody, WAKO; 1: 2000) overnight at 4°C. The rinsed sections were incubated secondary antibody (goat anti-rabbit IgG Alexa 488, Molecular Probes; 1:500) for 3 h at room temperature. Nuclear were counterstained with DAPI. Sections were mounted on slides and coverslipped by PermaFluor Aqueous mounting medium. Images were obtained using LSM510META (Carl Zeiss, Germany).
