# Supplementary material for: Hydrogen in Drinking Water Reduces Dopaminergic Neuronal Loss in the 1-methyl-4-phenyl-1,2,3,6-tetrahydropyridine Mouse Model of Parkinson's Disease
Source: PLoS One. 2009 Sep 30;4(9):e7247. doi: 10.1371/journal.pone.0007247 (PMC2747267; doi:10.1371/journal.pone.0007247)
Supplement: Materials and Methods S2 — (0.02 MB DOC) [file pone.0007247.s004.doc]

**Measurement of H_2_ contents in brain**

H_2_ contents in brain were measured by voltammetry using hydrogen electrode (teflon-coated platinum electrode with 2 mm bare tip; UNIQUE MEDICAL Co., LtD., Japan). Briefly, rats (Wister; Kyudo, Japan) were inserted guide-cannula into right striatum (-0.2 mm anterior, 3.5 mm lateral, 3.5 mm height from bregma) [27], [28]. One week after insertion, hydrogen electrode were inserted into guide-cannula to 1 or 2 mm ahead of the cannula. Anesthetized rats were inhaled H_2_ gas after the current became stable while voltage was clamped to -0.16V (vs. Ag/AgCl). In drinking H_2_ water, free-moving rats were received saturated H_2_ water after the insert of H_2_ electrode. Also, 5 mL of saturated H_2_ water was placed in stomach of anesthetized rats using catheter. Absolute H_2_ concentration was obtained from standard curves of different concentration of H_2_ water versus currents.
