# Supplementary material for: Hydrogen in Drinking Water Reduces Dopaminergic Neuronal Loss in the 1-methyl-4-phenyl-1,2,3,6-tetrahydropyridine Mouse Model of Parkinson's Disease
Source: PLoS One. 2009 Sep 30;4(9):e7247. doi: 10.1371/journal.pone.0007247 (PMC2747267; doi:10.1371/journal.pone.0007247)
Supplement: References S1 — (0.02 MB DOC) [file pone.0007247.s005.doc]

**S1.** Ifuku M, Färber K, Okuno Y, Yamakawa Y, Miyamoto T, et al. (2007) Bradykinin-induced microglial migration mediated by B1-bradykinin receptors depends on Ca^2+^ influx via reverse-mode activity of the Na^+^/Ca^2+^ exchanger. J Neurosci 27:13065-13073.
